# Mental Health Through the COVID-19 Quarantine: A Growth Curve Analysis on Italian Young Adults

**DOI:** 10.3389/fpsyg.2020.567484

**Published:** 2020-10-02

**Authors:** Anna Parola, Alessandro Rossi, Francesca Tessitore, Gina Troisi, Stefania Mannarini

**Affiliations:** ^1^Department of Humanities, University of Naples Federico II, Naples, Italy; ^2^Department of Philosophy, Sociology, Education and Applied Psychology, Section of Applied Psychology, University of Padua, Padua, Italy; ^3^Interdepartmental Centre for Family Research, University of Padua, Padua, Italy

**Keywords:** coronavirus disease 2019, quarantine, young adult, mental health, Achenbach adult self-report, internalizing/externalizing problems, growth model

## Abstract

**Introduction:**

Health emergencies, such as epidemics, have detrimental and long-lasting consequences on people’s mental health, which are higher during the implementation of strict lockdown measures. Despite several recent psychological researches on the coronavirus disease 2019 (COVID-19) pandemic highlighting that young adults represent a high risk category, no studies specifically focused on young adults’ mental health status have been carried out yet. This study aimed to assess and monitor Italian young adults’ mental health status during the first 4 weeks of lockdown through the use of a longitudinal panel design.

**Methods:**

Participants (*n* = 97) provided self-reports in four time intervals (1-week intervals) in 1 month. The Syndromic Scales of Adult Self-Report 18-59 were used to assess the internalizing problems (anxiety/depression, withdrawn, and somatic complaints), externalizing problems (aggressive, rule-breaking, and intrusive behavior), and personal strengths. To determine the time-varying effects of prolonged quarantine, a growth curve modeling will be performed.

**Results:**

The results showed an increase in anxiety/depression, withdrawal, somatic complaints, aggressive behavior, rule-breaking behavior, and internalizing and externalizing problems and a decrease in intrusive behavior and personal strengths from T1 to T4.

**Conclusions:**

The results contributed to the ongoing debate concerning the psychological impact of the COVID-19 emergency, helping to plan and develop efficient intervention projects able to take care of young adults’ mental health in the long term.

## Introduction

The novel coronavirus disease 2019 (COVID-19) is a highly infectious disease that began as a viral pneumonia in late December 2019. In March 2020, the World Health Organization (WHO) declared the state of pandemic.

As rapidly pointed out (Fiorillo and Gorwood, 2020; Jakovljevi et al., 2020), the COVID-19 global pandemic has affected—and is still affecting—not only physical health but also individual, family, and collective mental health. In line with recent studies (Horesh and Brown, 2020; Masiero et al., 2020), the COVID-19 pandemic should be classified as a critical event with a potential traumatic nature, which may be overwhelming and could lead to complex emotional responses that can negatively affect individuals and collective psychological systems.

Starting with China and followed by other states, extraordinary measures and containment efforts (e.g., lockdown) aimed to prevent the high risk of contagion and limit the COVID-19 outbreak have been adopted. In Europe, Italy was the first country that had to face the pandemic. Here, on March 09, 2020, strict lockdown measures were imposed by the government. A series of decrees imposed restrictions on the movements of individuals in the entire national territory from March 10 until May 3. During the lockdown, people were allowed to leave their homes only for limited and documented purposes. Schools, universities, theaters, and cinemas, as well as any shops selling non-essential goods were, therefore, temporarily closed.

As previous studies demonstrated (Tucci et al., 2017), health emergencies, such as epidemics, have detrimental and long-lasting consequences on people’s mental health. Concerning the COVID-19 pandemic, initial studies carried out in China reported high levels of anxiety, depression, and trauma-related symptoms (Qiu et al., 2020), both during the epidemic peak and 1 month later (Wang et al., 2020). Moreover, the detrimental effect of epidemics on mental health seems to be higher during the implementation of strict lockdown measures. Specifically, previous studies have associated quarantine with higher levels of trauma-related disorders (Wu et al., 2009), depression (Hawryluck et al., 2004), irritability and insomnia (Lee et al., 2005), acute stress (Bai et al., 2004), and avoidance behaviors and anger (Marjanovic et al., 2007). In a recent review, Brooks et al. (2020) individuated major stress factors as being the long duration of quarantine, the fear of infection, the inadequate supplies and information, boredom, and frustration. In a recent Italian study carried out during the third week of lockdown, Cellini et al. (2020) have highlighted that Italians reported high levels of depression, anxiety, and sleep disturbances. Similarly, Rossi A. et al. (2020) have found that high rates of negative mental health outcomes were seen in the general population 3 weeks into the COVID-19 lockdown.

Within the stream of research investigating the impact of quarantine during epidemics on individual’s mental health, there have been very few longitudinal investigations aimed at understanding and monitoring the changes in the mental health status during quarantine (Brooks et al., 2020). Where longitudinal research designs were carried out, they were limited to investigating people’s mental health during and after quarantine (Jeong et al., 2016; Wang et al., 2020).

Recent psychological research on COVID-19 has also highlighted that specific target groups are more at risk than others to develop a wide variety of psychological problems, such as medical workers, marginalized people (i.e., homeless and migrants), and young adults. Regarding young adults (18–30 years old), recent researches have highlighted that they present higher levels of anxiety, distress, and depression than do other adult groups (Cao et al., 2020; Huang and Zhao, 2020; Qiu et al., 2020). These findings have also been confirmed in Italy (Rossi R. et al., 2020). According to Cheng et al. (2014), one of the possible reasons can be found in young adults’ tendency to obtain information from social media, which can represent a high stress factor for mental health. These initial findings strongly suggest the need to assess and monitor young adults’ psychological situation during the epidemic and the weight of their mental health outcomes. To the best of the authors’ knowledge, there are no previous studies specifically aimed at evaluating the impact of lockdown measures on Italian young adults’ mental health and monitor the changes in their mental health status.

To fill this gap, the current study presents a longitudinal panel design aimed to assess the Italian young adults’ mental health status and monitor their mental health trends during the firsts 4 weeks of lockdown imposed from the Italian government during the COVID-19 outbreak. On the basis of recent literature on the general population, an increase in mental health problems among young adults during quarantine was hypothesized.

## Materials and Methods

### Participants

Participants were enrolled online and provided self-reports over 1 month (1-week intervals, T1–T2–T3–T4). Participants were considered eligible for participation if they met the following inclusion criteria: (a) were between 19 and 29 years and (b) were in a lockdown condition. Exclusion criteria were as follows: (a) diagnosis of psychiatric disorder and/or psychopharmacological treatment (assessed with filter questions in the survey) and (b) not “absolute” lockdown condition (workers who were allowed to work outside their home during the lockdown measures).

From the initial sample size of T1 (*N* = 120), nine participants did not participate at T2 (*N* = 111); four other participants did not participate at T3 (*N* = 107); and 10 other participants did not participate at T4. These participants were, therefore, excluded from the data analysis. The final simple-size was composed of 97 participants.

### Procedure

Approval from the University Research Ethics Committee was obtained for collecting data. Data collection took place during the Italian lockdown from mid-March 2020 to mid-April 2020. The administration took place in four time intervals (1-week intervals) in 1 month. The first survey (T1) was made at the end of the first week of lockdown. The second survey (T2) coincided with the end of the second week of the lockdown. The third survey (T3) coincided with the end of the third week of the lockdown. The fourth survey (T4) coincided with the end of the fourth week of the lockdown.

Participants were informed about a complete guarantee of confidentiality and the voluntary nature of participation and their right to discontinue at any point. The enrollment procedure was carried out through an online advertising on social platforms. Participants voluntarily accessed the online platform used for data collection once a week for the 4 weeks of administration. To ensure anonymity, a request was made to create a personal identification code to be used for the four administrations.

### Measures

#### Adult Self-Report (ASR/18-59)

The Syndromic Scales of Adult Self-Report 18-59 (Achenbach and Rescorla, 2003) were used to assess the internalizing and externalizing problems.

The ASR is especially valuable when used routinely, as in this study design. The ASR norms provide a standardized benchmark with which to compare what is reported by each individual. Standardized reassessments over a regular interval enable to identify reported stabilities and changes in a group who have particular kinds of problems. In this case, the ASR instrument was administered at regular intervals of 1 week for 4 weeks in the period of the Italian lockdown. The ASR was developed both to document specific problems and to identify syndromes of co-occurring problems. In this study, six specific Syndromic Scales, Anxious/Depressed, Withdrawn, Somatic Complaints, Aggressive Behavior, Rule-Breaking Behavior, and Intrusive were used. Anxious/Depressed (18 items) refers to anxiety and depressive symptoms (e.g., “I feel lonely” and “I am too fearful or anxious”). Withdrawn (8 items) mainly refers to attitudes of isolation and lack of contact with others (e.g., “I don’t get along with other people” and “I keep from getting involved with others”). Somatic Complaints (12 items) include physical illness, without a known medical cause (e.g., “I feel dizzy or lightheaded” and “Physical problems without a known medical cause: stomachaches”). Aggressive Behavior (15 items) includes behaviors and attitudes characterized by poor control of one’s aggression (e.g., “I blame others for my problems” and “I scream or yell a lot”). Rule-Breaking Behavior (14 items) refers to transgressive behavior and violation of social norms (e.g., “I am impulsive or act without thinking” and “I lie or cheat”). Intrusive (6 items) refers to the difficulty faced in the interpersonal relationships and to the prevalence of intrusive behavior (e.g., “I damage or destroy my things” and “I drink too much alcohol or get drunk”). In addition, the broadband scales, Internalizing and Externalizing, were computed. Internalizing problems reflect internal distress, while externalizing problems reflect conflicts with other people. The Internalizing scale consists of the syndrome scales Anxious/Depressed, Withdrawn, and Somatic Complaints, whereas the Externalizing scale consists of Aggressive Behavior and Rule-Breaking Behavior. Moreover, the scale of Personal Strengths (11 items) was used to assess the adaptive functioning of the individuals (e.g., “I try to get a lot of attention” and “I am louder than others”).

The items are scored on a three-point rating scale: 0 (*not true*), 1 (*somewhat or sometimes true*), and 2 (*very true or often true*); and a total score may be calculated. Higher raw scores indicate more problematic behaviors on each scale. Then, a normalized *T* score—weighted for sex and age—was assigned for the Syndromic Scales and to each Internalizing and Externalizing Problem scales. Raw scores of the both types of scales have been quantitatively converted in terms of gender- and age-specific *T* scores. Clinical significant threshold is indicated by *T*-scores ≥ 70. Borderline range is from 65 to 69.

The ASR is a reliable and valid measure for the 18–59 general population (Achenbach and Rescorla, 2003). Cronbach’s alpha (α) and McDonald’s omega (ω) are reported in Table 1.

**TABLE 1:** Mean, standard deviation, reliability coefficients, and effect size (|g|) for each time comparison.

		Descriptive		Reliability		Time comparison (Hedge’s *g*)			
		*M*	*SD*	α	ω	T1	T2	T3	T4
**Anxious/depressed**									
1	T1	58.40	8.61	0.88	0.91	–			
2	T2	61.82	9.39	0.88	0.90	0.38	–		
3	T3	70.64	15.28	0.94	0.96	0.98	0.69	–	
4	T4	69.34	13.70	0.92	0.93	0.95	0.64	0.09	–
**Withdrawn**									
1	T1	58.82	9.23	0.81	0.87	–			
2	T2	59.23	9.17	0.80	0.86	0.26	–		
3	T3	65.70	15.67	0.93	0.95	0.69	0.50	–	
4	T4	66.64	15.53	0.91	0.93	0.76	0.58	0.06	–
**Somatic complaints**									
1	T1	55.16	6.75	0.72	0.80	–			
2	T2	57.72	8.46	0.77	0.82	0.33	–		
3	T3	58.36	8.40	0.81	0.86	0.42	0.08	–	
4	T4	58.26	8.44	0.81	0.87	0.40	0.06	0.01	–
**Aggressive behaviors**									
1	T1	55.29	6.43	0.90	0.89	–			
2	T2	57.61	7.12	0.87	0.91	0.34	–		
3	T3	61.33	10.74	0.91	0.95	0.68	0.41	–	
4	T4	61.33	10.66	0.91	0.94	0.68	0.41	0.00	–
**Rule-breaking behavior**									
1	T1	53.61	5.01	0.68	0.75	–			
2	T2	54.62	6.82	0.82	0.87	0.17	–		
3	T3	57.22	6.20	0.65	0.79	0.17	0.40	–	
4	T4	57.60	6.25	0.64	0.78	0.72	0.45	0.06	–
**Intrusive**									
1	T1	54.80	6.48	0.75	0.84	–			
2	T2	54.87	6.09	0.64	0.81	0.01	–		
3	T3	53.27	4.55	0.65	0.71	0.27	0.30	–	
4	T4	53.23	4.25	0.63	0.68	0.28	0.31	0.01	–
**Internalizing scales**									
1	T1	55.33	11.32	0.91	0.93	–			
2	T2	60.32	10.62	0.91	0.93	0.45	–		
3	T3	67.26	13.01	0.65	0.75	0.97	0.58	–	
4	T4	66.95	12.20	0.78	0.88	0.98	0.58	0.02	–
**Externalizing scales**									
1	T1	51.71	9.14	0.87	0.90	–			
2	T2	54.44	9.76	0.90	0.92	0.29	–		
3	T3	58.26	9.02	0.88	0.92	0.72	0.40	–	
4	T4	58.41	9.03	0.85	0.90	0.74	0.42	0.02	–
**Personal strengths**									
1	T1	16.79	2.64	0.66	0.70	–			
2	T2	16.08	2.68	0.65	0.75	0.27	–		
3	T3	15.30	4.02	0.85	0.90	0.44	0.23	–	
4	T4	15.10	2.40	0.83	0.89	0.67	0.38	0.06	–

### Data Analysis

Statistical analyses were performed with R software (v. 3.5.3; R Core Team, 2014, 2015) and the following packages: psych (v. 1.8.12; Revelle, 2018), irr (v. 0.84.1; Gamer et al., 2019), lme4 (v.1.1-21; Bates et al., 2015), lmerTest (v. 3.1-2; Kuznetsova et al., 2017), esvis (v. 0.3.1; Anderson, 2020), AICcmodavg (v2.3-0; Mazerolle, 2020), and ggplot2 (v. 3.1.0; Wickham, 2016).

No data were missing for any of the participants on any of the ASR scales at any of the measurement points. Reliability was evaluated by internal consistency analysis, using Cronbach’s alpha (α) and McDonald’s omega for categorical data (ω).

First, the mean differences between the four time intervals (T1, T2, T3, and T4) were performed. The unbiased sample estimate of standardized mean difference effect sizes (Hedges’ *g*; Hedges, 1981) was performed, evaluating the magnitude of these differences. The following established ranges guide interpreting standardized mean difference magnitude: from 0.20 to 0.49 = small; from 0.50 to 0.79 = medium; and 0.80 = large (Cohen, 1988).

Growth curve analysis (GCA) models were used to estimate the growth trajectories (i.e., slopes) of the Syndromic Scales of the ASR—both Internalizing and Externalizing scales—and the personal strength scale. Models also estimated subject variability in change across time, as represented in random-intercepts coefficients. Parameters in each GCA model were computed with maximum likelihood (ML) estimation.

Several models were estimated for each of the outcome variables, separately. Specifically, it was hypothesized that the time (the week of quarantine) could have had an effect on the ASR Syndromic Scales. In addition, it was also hypothesized that covariates, such as sex and the experience of COVID-19 (EXP-CVD19), intended as the experience of direct proximity with relatives and/or friends affected by COVID-19, could have had an effect on the shape of the growth curve across time. Models were sequentially specified according to the guidelines (Long, 2012; Grimm et al., 2017). *First*, a null model was estimated to provide a baseline comparison and to calculate the intraclass correlation coefficient (Model 0—Intercept only). *Second*, a null model with covariates was specified (Model 1—Intercept model with covariates). *Third*, a linear model with time as predictor and covariate interactions was estimated (Model 2—Linear model with covariates). *Fourth*, a quadratic model was specified with linear interaction effects of the covariates (Model 3—Quadratic model with linear covariates interactions). *Fifth*, a quadratic model was specified with all possible interactions of the covariates (Model 4—full quadratic model with covariates). Equations of each model are reported in Table 2.

**TABLE 2:** Equations of each estimated model.

Model		Equation
M.0	Intercept only	*y*_ij_ = (β_0_ + *b*_0i_) + ε_ij_

M.1	Intercept model with covariates	*y*_ij_ = (β_0_ + *b*_0i_) + β_1_(*sex*_i_) + β_2_(*experience with COVID*19_i_) + ε_ij_

M.2	Linear model with covariates	*y*_ij_ = (β_0_ + *b*_0i_) + β_1_(*week of quarantine*_*i*j_) + β_2_(*sex*_i_) + β_3_(*experience with COVID*19_i_) + β_4_(*week of quarantine*_ij_ * *sex*_i_)
		+ β_5_(*week of quarantine*_ij_ * *experience with COVID*19_i_) + ε_ij_

M.3	Quadratic model with linear covariates interactions	$y_{\text{ij}}=\left(\beta_{0}+b_{0\,i}\right)+\beta_{1}\,\left(w\,e\,e\,k\,o\,f\,q\,u\,a\,r\,a\,n\,t\,i\,n\,e_{\text{ij}}\right)+\beta_{2}\,\left(w\,e\,e\,k\,o\,f\,q\,u\,a\,r\,a\,n\,t\,i\,n\,e_{\text{ij}}^{2}\right)+\beta_{3}\,\left(s\,e\,x_{\text{i}}\right)+\beta_{4}\,\left(e\,x\,p\,e\,r\,i\,e\,n\,c\,e\,w\,i\,t\,h\,C\,O\,V\,I\,D\,19_{\text{i}}\right)$
		+ β_5_(*week of quarantine*_ij_ * *sex*_i_) + β_6_(*week of quarantine*_ij_ * *experience with COVID*19_i_) + ε_ij_

M4	Quadratic model with all covariates interactions	$y_{\text{ij}}=\left(\beta_{0}+b_{0\,i}\right)+\beta_{1}\,\left(w\,e\,e\,k\,o\,f\,q\,u\,a\,r\,a\,n\,t\,i\,n\,e_{\text{ij}}\right)+\beta_{2}\,\left(w\,e\,e\,k\,o\,f\,q\,u\,a\,r\,a\,n\,t\,i\,n\,e_{\text{ij}}^{2}\right)+\beta_{3}\,\left(s\,e\,x_{\text{i}}\right)+\beta_{4}\,\left(e\,x\,p\,e\,r\,i\,e\,n\,c\,e\,w\,i\,t\,h\,C\,O\,V\,I\,D\,19_{\text{i}}\right)$
		+ β_5_(*week of quarantine*_ij_ * *sex*_i_) + β_6_(*week of quarantine*_ij_ * *experience with COVID*19_i_)
		$+\beta_{7}\,\left(w\,e\,e\,k\,o\,f\,q\,u\,a\,r\,a\,n\,t\,i\,n\,e_{\text{ij}}^{2}*s\,e\,x_{\text{i}}\right)+\beta_{8}\,\left(w\,e\,e\,k\,o\,f\,q\,u\,a\,r\,a\,n\,t\,i\,n\,e_{\text{ij}}^{2}*e\,x\,p\,e\,r\,i\,e\,n\,c\,e\,w\,i\,t\,h\,C\,O\,V\,I\,D\,19_{\text{i}}\right)+\varepsilon_{\text{ij}}$

The best model fit was assessed with several indices. First of all, the likelihood ratio test (LRT) was performed between one model and the following one in a step-up approach analysis: Model 0 vs. Model 1; Model 1 vs. Model 2; Model 2 vs. Model 3; and Model 3 vs. Model 4—the most parsimonious model will be preferred (Long, 2012). In addition, also “information criteria” indices were computed by comparing the abovementioned models. First, the Schwarz Bayesian information criterion (BIC; Schwarz, 1978; Burnham and Anderson, 2002) was calculated: the model with the lower BIC indicated the best model—and it is recommended when model parsimony is overriding (Kadane and Lazar, 2004; Long, 2012). Moreover, considering that the BIC tends to favor simpler model (Long, 2012), the corrected Akaike information criterion (AICc; Akaike, 1973; Azari et al., 2006) was also computed: even in this case, the model with the lower AICc indicated the best model. In addition, considering that—on a theoretical level—the BIC is less desirable for model evaluation than the AICc (Long, 2012), several effect sizes based on the AICc were carried out: (I) the difference of AICc (ΔAICc); (II) the weight of evidence (*W*_h_): given a set of competing models and the unknowable true model, the *W*_h_ indicates the probability that a model *h* is the best approximate model (the model with the large *W*_h_ is the best-fitting model) (the more probable the model is, the best approximating the model will be to the true model); (III) the evidence ratio (*E*_h_) that expresses the difference—in odds—between the best-fitting model and the first worst-fitting model: the higher the *E*_h_, the more plausible is the best-fitting model.

## Results

### Preliminary Analysis

Of 97 participants, 48 were male (49.5%) and 49 were female (50.5%). The mean age of the sample was 24.62 (*SD* = 2.88; range = 19–29). A total of 29 participants (29.9%) had experienced proximity with a COVID-19-infected relative or friend. Most of the participants lived with their parents during the quarantine (80.4%). All participants came from the Campania region, in Southern Italy, and attended the university.

Means and standard deviations between the four time intervals (T1, T2, T3, and T4) and the effect size of means difference (Hedges’ *g*) are displayed in Table 1. The preliminary analysis showed that the increments tended to be small from T1 to T2 for each syndromic scale and breadboard scale (0.45 was the highest value). From T2 to T3, the results highlighted a medium increase for the Anxious/Depressed, Withdrawn, and Internalizing scales. From T3 to T4, the increase was null. For Somatic Complaints, Aggressive Behaviors, Rule-Breaking Behavior, and Externalizing scales, the magnitude of the effect size was medium only considering the increments from T1 to T4. Across the weeks of quarantine, the Somatic Complaints scale increased with an almost null effect. Finally, the Personal Strengths showed a small increase only from T1 to T3 and from T1 to T4.

**FIGURE 1:**
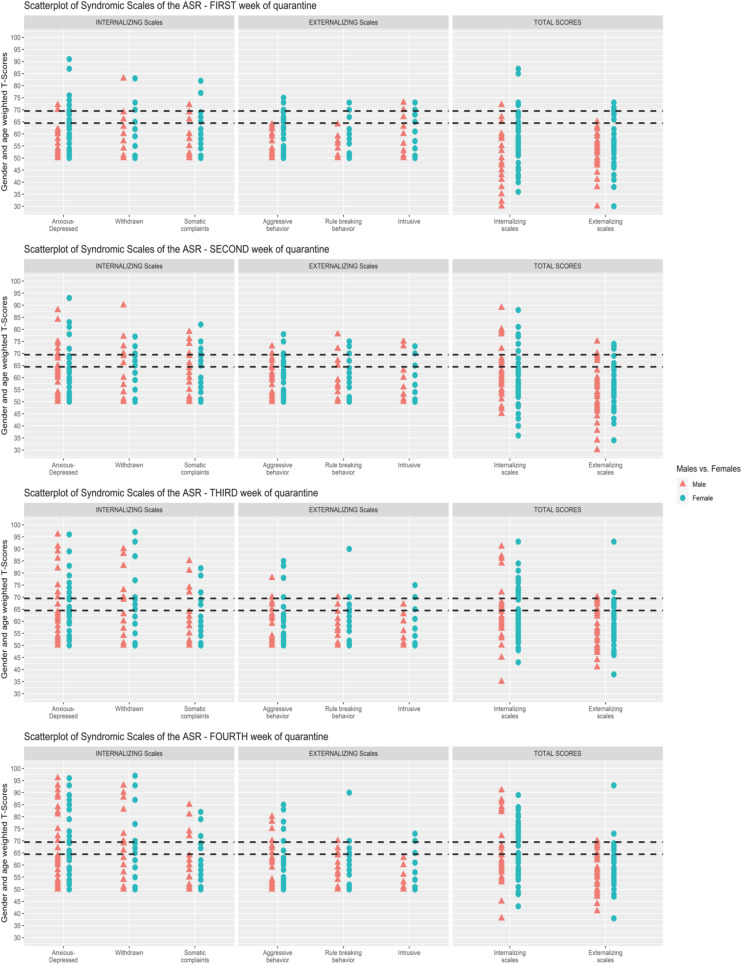
Scatterplot of Syndromic Scales of the Adult Self-Report (ASR) for each week of quarantine.

**FIGURE 2:**
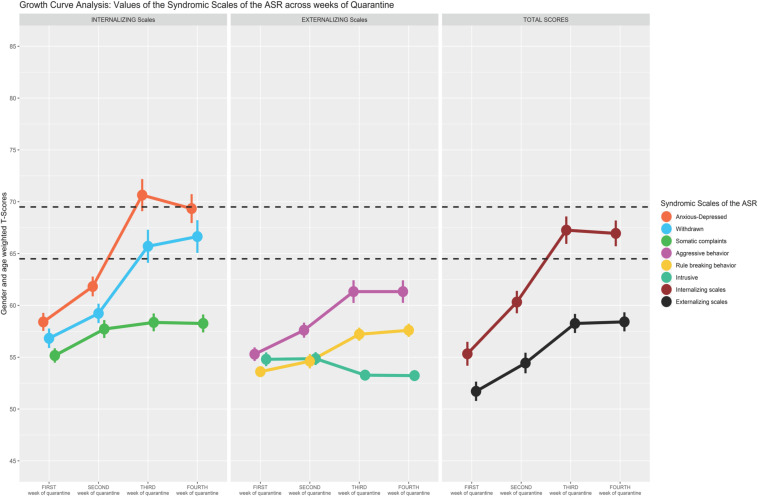
Growth curve analysis: means and standard error of the Syndromic Scale across weeks of quarantine.

**FIGURE 3:**
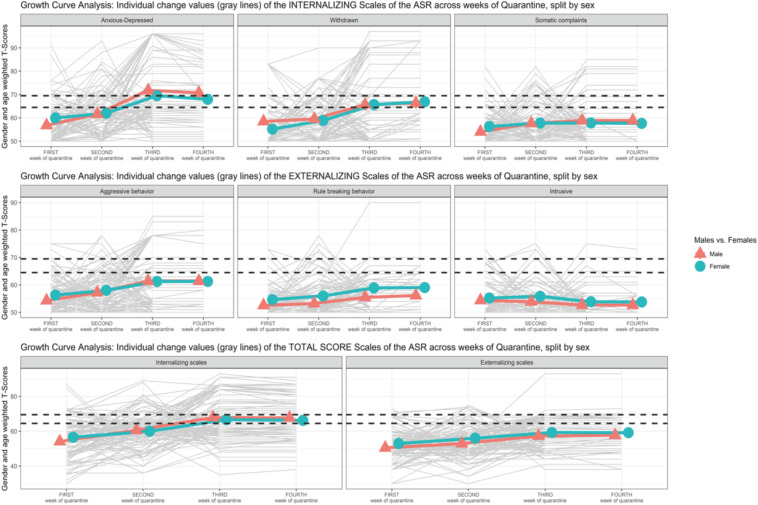
Growth curve analysis (GCA): means and standard error of the Syndromic Scale across weeks of quarantine split by sex.

**FIGURE 4:**
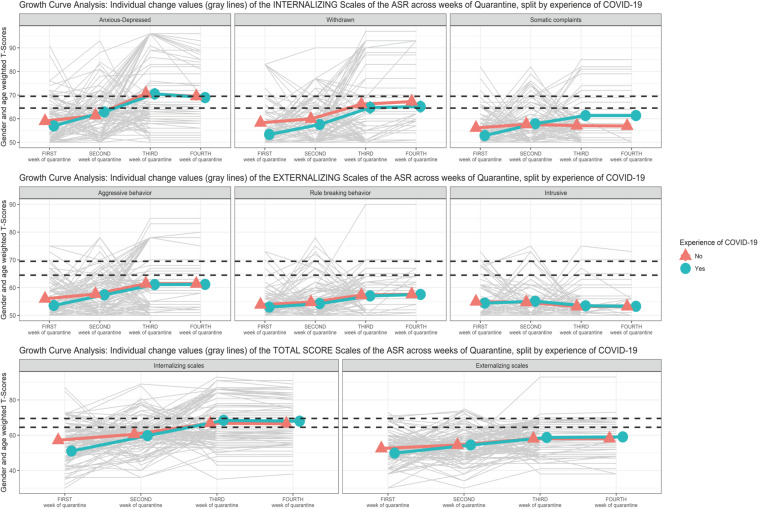
Growth curve analysis (GCA): means and standard error of the Syndromic Scale across weeks of quarantine split by “experience of COVID-19”.

**FIGURE 5:**
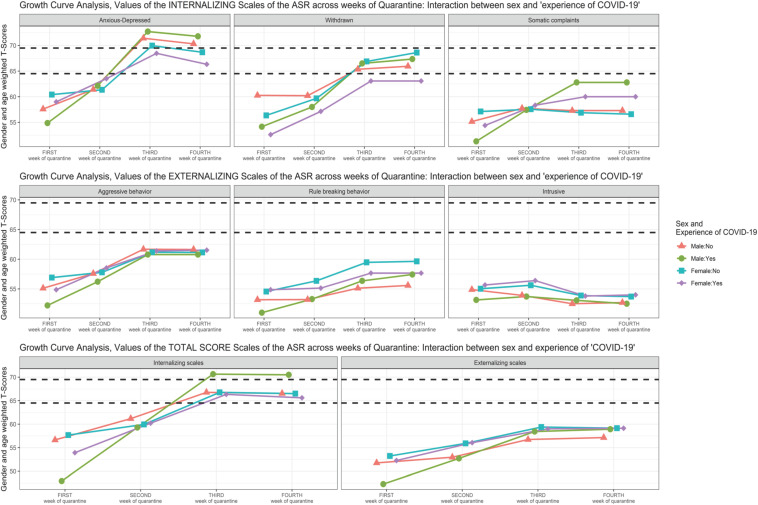
Growth curve analysis (GCA): means of the Syndromic Scale across weeks of quarantine—interaction between “sex” and “experience of COVID-19”.

Scatterplot (Figure 1) showing the change of the Syndromic Scales and broadband scales score over time. Figures 2–5 graphically show means and standard error of the Syndromic Scales, as well as the related broadband scales, across the weeks of quarantine. Specifically, Figure 3 was split by sex (males vs. females), and Figure 4 was split by the experience of COVID-19 (yes vs. no). Finally, Figure 5 shows the interaction between sex and experience of COVID-19. The broken lines demarcate a borderline clinical range from the 93rd to 97th percentiles for the Syndromic scales and from the 84th to 90th percentiles for the Internalizing and Externalizing broadband scales. Scores above the top broken line, i.e., above the 97th percentile for the Syndromic scales and above the 90th for the Internalizing and Externalizing broadband, indicate that the individual reported enough problems to be of clinical concern. Scores below the bottom broken line is in the normal range. As show in Figure 2, the Anxious/Depressed scale is above the clinical threshold in T3, and the Withdrawn scale is above the normal threshold in T3 with an increase in T4.

### Anxious/Depressed

Preliminary analyses (M.0) revealed that the variance related to the random intercept of the participants was equal to 24.41.

The null model with covariates (M.1) revealed a non-statistically significant effect of the interaction between sex and EXP-CVD19 (*b* = −0.961, *SE* = 3.460, *t* = 0.077, *p* = 0.782) or their main effects (sex: *b* = −0.081, *SE* = 1.891, *t* = −0.043, *p* = 0.966; EXP-CVD19: *b* = 0.191, *SE* = 2.476, *t* = 0.077, *p* = 0.939).

The linear model with covariates (M.2) revealed a non-statistically significant effect of the interaction between time and EXP-CVD19 (*b* = 0.310, *SE* = 1.065, *t* = 0.291, *p* = 0.771) or the two simple main effects (sex: *b* = 4.797, *SE* = 2.908, *t* = 1.649, *p* = 0.099; EXP-CVD19: *b* = −1.076, *SE* = 3.176, *t* = −0.339, *p* = 0.735). However, the model revealed a statistically significant interaction effect between time and sex (*b* = −2.066, *SE* = 0.975, *t* = −2.118, *p* = 0.035) as well as the principal effect of time (*b* = 5.114, *SE* = 0.760, *t* = 6.732, *p* < 0.001). Figure 3 shows a greater increase in males from T1 to T2 and from T2 to T3 than in females.

The quadratic model with linear interaction (M.3) showed a non-statistically significant effect of the interaction between time and EXP-CVD19 (*b* = 0.310, *SE* = 1.057, *t* = 0.293, *p* = 0.769) or the two simple main effects (sex: *b* = 4.797, *SE* = 2.892, *t* = 1.659, *p* = 0.098; EXP-CVD19: *b* = −1.076, *SE* = 3.156, *t* = −0.341, *p* = 0.734). However, the model revealed a statistically significant linear interaction effect between time and sex (*b* = −2.066, *SE* = 0.967, *t* = −2.125, *p* = 0.033) as well as the principal effect of time: both linear (*b* = 11.016, *SE* = 2.807, *t* = 3.925, *p* < 0.001) and quadratic (*b* = −1.180, *SE* = 0.541, *t* = −2.183, *p* = 0.030).

Finally, the quadratic model with all covariates interactions (M.4) showed a non-statistically significant effect of the interaction between time and EXP-CVD19, neither linear (*b* = 5.1607, *SE* = 5.991, *t* = 0.861, *p* = 0.340) nor quadratic (*b* = −0.970, *SE* = 1.179, *t* = −0.823, *p* = 0.411). In addition, M.4 showed also a non-statistically significant effect of the interaction between time and sex, neither linear (*b* = −4.990, *SE* = 5.485, *t* = −0.910, *p* = 0.364) nor quadratic (*b* = 0.585, *SE* = 1.080, *t* = 0.542, *p* = 0.589). Furthermore, also the simple main effects of sex (*b* = −7.721, *SE* = 6.123, *t* = 1.261, *p* = 0.208), EXP-CVD (*b* = −5.926, *SE* = 6.688, *t* = −0.886, *p* = 0.376), and time (quadratic term: *b* = −1.186, *SE* = 0.841, *t* = −1.410, *p* = 0.160) revealed a non-statistically significant effect. Only the main effect of time (linear term: *b* = 11.043, *SE* = 4.272, *t* = 2.585, *p* = 0.010) became statistically significant.

The comparison of the different multilevel growth curve models provided ambiguous results. Indeed, M.2 showed the lower BIC, but M.3 showed the lower AICc. The LRT suggested a statistical significant difference between M.3 and M.2 [χ^2^(1) = 4.726; *p* = 0.030]. In addition, the ΔAICc suggested a small difference M.3 and M.2 (2.63); the *W*_h_ of M.3 suggested that this model had 68% probability of being the best approximate model; and the *E*_h_ suggested that M.3 had a weight of evidence almost four times (3.72) greater than that of M.2 of being the best approximate model (Table 3).

**TABLE 3:** Model comparisons for each ASR scale.

		Log likelihood	LRT: χ^2^ (*df*)	*p*-value	BIC	AICc	ΔAICc	*W*_h_
**Anxious/depressed**								
M.0	Intercept only	−1,542.2			3,102.3	3,090.5	61.06	0.00
M.1	Intercept model with covariates	−1,542.1	0.163 (3)	0.983	3,120.0	3,096.5	67.05	0.00
M.2	Linear model with covariates	−1,507.8	68.581 (2)	<0.001	3,063.4	3,032.1	2.63	0.18
M.3	Quadratic model with linear covariates interactions	−1,505.5	4.726 (1)	0.030	3,064.6	3,029.4	BM	0.68
M.4	Quadratic model with all covariates interactions	−1,505.0	0.955 (2)	0.621	3,075.6	3,032.7	3.27	0.13
**Withdrawn**								
M.0	Intercept only	−1,546.4			3,110.7	3,098.9	42.44	0.00
M.1	Intercept model with covariates	−1,545.1	2.697 (3)	0.441	3,125.9	3,102.3	45.90	0.00
M.2	Linear model with covariates	−1,520.0	50.064 (2)	<0.001	3,087.7	3,056.4	BM	0.66
M.3	Quadratic model with linear covariates interactions	−1,519.8	0.439 (1)	0.507	3,093.3	3,058.1	1.66	0.29
M.4	Quadratic model with all covariates interactions	−1,519.5	0.686 (2)	0.710	3,104.5	3,061.6	5.20	0.05
**Somatic complaints**								
M.0	Intercept only	−1,360.3			2,783.5	2,726.7	17.11	0.00
M.1	Intercept model with covariates	−1,359.3	2.031 (3)	0.566	2,754.3	2,730.8	21.24	0.00
M.2	Linear model with covariates	−1,347.1	24.340 (2)	<0.001	2,741.9	2,710.6	1.06	0.32
M.3	Quadratic model with linear covariates interactions	−1,345.5	3.152 (1)	0.076	2,744.7	2,709.6	BM	0.54
M.4	Quadratic model with all covariates interactions	−1,344.8	1.480 (2)	0.477	2,755.2	2,712.3	2.75	0.14
**Aggressive behaviors**								
M.0	Intercept only	−1,407.7			2,833.2	2,821.4	26.57	0.00
M.1	Intercept model with covariates	−1,407.2	1.009 (3)	0.799	2,850.1	2,826.6	21.72	0.00
M.2	Linear model with covariates	−1,389.2	35.880 (2)	<0.001	2,826.2	2,794.9	BM	0.47
M.3	Quadratic model with linear covariates interactions	−1,388.2	2.077 (1)	0.149	2,830.1	2,794.9	0.02	0.46
M.4	Quadratic model with all covariates interactions	−1,388.0	0.495 (2)	0.781	2,841.5	2,798.6	3.75	0.07
**Rule-breaking behaviors**								
M.0	Intercept only	−1,260.0			2,537.9	2,526.1	37.00	0.00
M.1	Intercept model with covariates	−1,252.2	15.723 (3)	0.001	2,540.1	2,516.5	27.43	0.00
M.2	Linear model with covariates	−1,236.4	31.591 (2)	<0.001	2,520.4	2,489.1	BM	0.68
M.3	Quadratic model with linear covariates interactions	−1,236.2	0.314 (1)	0.575	2,526.1	2,490.9	1.78	0.28
M.4	Quadratic model with all covariates interactions	−1,236.1	0.271 (2)	0.873	2,537.7	2,494.9	5.74	0.04
**Intrusive**								
M.0	Intercept only	−1,207.0			2,431.9	2,420.1	1.44	0.23
M.1	Intercept model with covariates	−1,204.6	4.726 (3)	0.193	2,445.1	2,421.5	2.87	0.11
M.2	Linear model with covariates	−1,201.1	7.029 (2)	0.030	2,450.0	2,418.6	BM	0.47
M.3	Quadratic model with linear covariates interactions	−1,201.1	0.010 (1)	0.921	2,455.9	2,420.7	2.09	0.17
M.4	Quadratic model with all covariates interactions	−1,200.9	0.526 (2)	0.769	2,467.3	2,424.4	5.79	0.03
**Internalizing scales**								
M.0	Intercept only	−1,536.1			3,090.1	3,078.2	65.61	0.00
M.1	Intercept model with covariates	−1,535.9	0.398 (3)	0.941	3,107.6	3,084.0	71.37	0.00
M.2	Linear model with covariates	−1,500.1	71.621 (2)	<0.001	3,047.9	3,016.5	3.91	0.10
M.3	Quadratic model with linear covariates interactions	−1,497.1	6.002 (1)	0.014	3,047.8	3,012.6	BM	0.69
M.4	Quadratic model with all covariates interactions	−1,496.2	1.845 (2)	0.397	3,057.9	3,015.0	2.38	0.21
**Externalizing scales**								
M.0	Intercept only	−1,427.5			2,872.9	2,861.1	30.32	0.00
M.1	Intercept model with covariates	−1,425.1	4.710 (3)	0.194	2,886.1	2,862.5	31.77	0.00
M.2	Linear model with covariates	−1,407.2	35.908 (2)	<0.001	2,862.1	2,830.8	0.02	0.46
M.3	Quadratic model with linear covariates interactions	−1,406.1	2.113 (1)	0.146	2,865.9	2,830.7	BM	0.47
M.4	Quadratic model with all covariates interactions	−1,405.9	0.491 (2)	0.782	2,877.3	2,834.5	3.73	0.07
**Personal strengths**								
M.0	Intercept only	−980.8			1,979.5	1,967.6	12.55	0.00
M.1	Intercept model with covariates	−980.1	1.350 (3)	0.717	1,996.0	1,972.4	17.36	0.00
M.2	Linear model with covariates	−969.3	21.515 (2)	<0.001	1,986.4	1,955.1	BM	0.61
M.3	Quadratic model with linear covariates interactions	−968.9	0.848 (1)	0.357	1,991.5	1,956.3	1.25	0.33
M.4	Quadratic model with all covariates interactions	−968.5	0.887 (2)	0.642	2,002.5	1,959.7	4.59	0.06

*BM, best model; ASR, adult self-report; LRT, likelihood ratio test; BIC, Bayesian information criterion; AICc, corrected Akaike information criterion; Wh, weight of evidence.*

### Withdrawn

Preliminary analyses (M.0) revealed that the variance related to the random intercept of the participants was equal to 38.95.

The null model with covariates (M.1) revealed a non-statistically significant effect of the interaction between sex and EXP-CVD19 (*b* = −2.474, *SE* = 3.768, *t* = −0.657, *p* = 0.513) or their main effects (sex: *b* = −0.059, *SE* = 2.059, *t* = −0.029, *p* = 0.977; EXP-CVD19: *b* = −1.441, *SE* = 2.696, *t* = −0.534, *p* = 0.594).

The linear model with covariates (M.2) revealed a non-statistically significant effect of the interaction between time and EXP-CVD19 (*b* = 0.925, *SE* = 1.079, *t* = 0.858, *p* = 0.392) or the two simple main effects (sex: *b* = −3.817, *SE* = 3.015, *t* = −1.266, *p* = 0.206; EXP-CVD19: *b* = −5.022, *SE* = 3.292, *t* = −1.525, *p* = 0.128). Moreover, the model revealed a non-statistically significant interaction effect between time and sex (*b* = 1.205, *SE* = 0.988, *t* = 1.222, *p* = 0.222). Only the main effect of time (*b* = 2.705, *SE* = 0.769, *t* = 3.515, *p* < 0.001) became statistically significant.

The quadratic model with linear interaction (M.3) showed a non-statistically significant effect of the interaction between time and EXP-CVD19 (*b* = 0.925, *SE* = 1.078, *t* = 0.858, *p* = 0.391) or the two simple main effects (sex: *b* = 3.817, *SE* = 3.013, *t* = −1.267, *p* = 0.206; EXP-CVD19: *b* = −5.022, *SE* = 3.291, *t* = −1.526, *p* = 0.128). Moreover, the model revealed a non-statistically significant linear interaction effect between time and sex (*b* = 1.207, *SE* = 0.987, *t* = 1.223, *p* = 0.222) as well as the principal effect of time: both linear (*b* = 4.535, *SE* = 2.864, *t* = 1.583, *p* = 0.114) and quadratic (*b* = −0.366, *SE* = 0.552, *t* = −0.663, *p* = 0.508).

Finally, the quadratic model with all covariates interactions (M.4) showed a non-statistically significant effect of the interaction between time and EXP-CVD19, neither linear (*b* = 5.035, *SE* = 6.116, *t* = 0.823, *p* = 0.411) nor quadratic (*b* = −0.822, *SE* = 1.204, *t* = −0.683, *p* = 0.495). In addition, M.4 showed also a non-statistically significant effect of the interaction between time and sex, neither linear (*b* = 3.739, *SE* = 5.600, *t* = −0.669, *p* = 0.505) nor quadratic (*b* = 0.506, *SE* = 1.102, *t* = −0.459, *p* = 0.646). Furthermore, also the simple main effects of sex (*b* = −6.349, *SE* = 6.281, *t* = −1.011, *p* = 0.313), EXP-CVD (*b* = −9.132, *SE* = 6.859, *t* = −1.331, *p* = 0.184), and time (linear term: *b* = 2.027, *SE* = 4.361, *t* = 0.465, *p* = 0.642, and quadratic term: *b* = −0.136, *SE* = 0.859, *t* = 0.158, *p* = 0.875) revealed a non-statistically significant effect.

The comparison of the different multilevel growth curve models suggested that the linear model with covariates (M.2) showed the lower BIC and the lower AICc. The LRT showed that M.2 was statistically significantly different from M.1 (intercept model with covariates). However, despite that M.2 was not statistically significantly different from M.3, it was the most parsimonious, and thus, it was chosen as the best model. Indeed, the ΔAICc suggested a small difference M.2 and M.3 (1.66), the *W*_h_ of M.2 indicates that this model had 66% probability of being the best approximate model, and the *E*_h_ suggested that M.2 had a weight of evidence more than two times (2.29) greater than M.3 of being the best approximate model (Table 3).

### Somatic Complaints

Preliminary analyses (M.0) revealed that the variance related to the random intercept of the participants was equal to 6.25.

The null model with covariates (M.1) revealed a non-statistically significant effect of the interaction between sex and EXP-CVD19 (*b* = −0.557, *SE* = 2.019, *t* = −0.276, *p* = 0.783) or their main effects (sex: *b* = 0.169, *SE* = 1.103, *t* = 0.153, *p* = 0.878; EXP-CVD19: *b* = 1.704, *SE* = 1.445, *t* = 1.179, *p* = 0.241).

The linear model with covariates (M.2) revealed a statistically significant effect of the interaction between time and EXP-CVD19 (*b* = 2.714, *SE* = 0.734, *t* = 3.698, *p* < 0.001), and of the simple main effect of EXP-CVD19 (*b* = −5.365, *SE* = 2.094, *t* = −2.562, *p* = 0.011). However, the model revealed a non-statistically significant main effect of sex (*b* = 3.049, *SE* = 1.912, *t* = 1.590, *p* = 0.113). Moreover, the model revealed a non-statistically significant interaction effect between time and sex (*b* = −1.219, *SE* = 0.762, *t* = 1.183, *p* = 0.070) as well as the principal effect of time (*b* = 0.796, *SE* = 0.523, *t* = 1.521, *p* = 0.129). Figure 4 shows a greater increase from T3 to T4 of the participants with EXP-CVD19.

The quadratic model with linear interaction (M.3) showed a statistically significant effect of the interaction between time and EXP-CVD19 (*b* = 2.714, *SE* = 0.729, *t* = 3.718, *p* < 0.001), and of the simple main effect of EXP-CVD19 (*b* = −5.365, *SE* = 2.085, *t* = −2.573, *p* = 0.010). However, the model revealed a non-statistically significant main effect of sex (*b* = 3.049, *SE* = 1.901, *t* = 1.597, *p* = 0.111). Moreover, the model revealed a non-statistically significant linear interaction effect between time and sex (*b* = −1.219, *SE* = 0.668, *t* = 1.823, *p* = 0.069). The model showed a statistically significant effect of time as linear (*b* = 4.121, *SE* = 1.938, *t* = 2.125, *p* = 0.034) but not as a quadratic term (*b* = −0.665, *SE* = 0.373, *t* = 1.780, *p* = 0.076).

Finally, the quadratic model with all covariates interactions (M.4) showed a non-statistically significant effect of the interaction between time and EXP-CVD19, neither linear (*b* = 6.929, *SE* = 4.134, *t* = 1.676, *p* = 0.094) nor quadratic (*b* = −0.843, *SE* = 0.814, *t* = −1.036, *p* = 0.301). In addition, M.4 showed also a non-statistically significant effect of the interaction between time and sex, neither linear (*b* = −3.668, *SE* = 3.786, *t* = −0.969, *p* = 0.333) nor quadratic (*b* = 0.490, *SE* = 0.745, *t* = 0.657, *p* = 0.511). Furthermore, also the simple main effects of sex (*b* = 5.498, *SE* = 4.186, *t* = 1.314, *p* = 0.190) and time (linear term: *b* = 4.098, *SE* = 2.948, *t* = 1.390, *p* = 0.166, and a quadratic term: *b* = −0.660, *SE* = 0.580, *t* = 1.138, *p* = 0.256) revealed a non-statistically significant effect. However, the model showed a statistically significant effect of EXP-CVD19 (*b* = −9.580, *SE* = 4.571, *t* = −2.096, *p* = 0.037).

The comparison of the different multilevel growth curve models provided unclear results. Indeed, the linear model (M.2) showed the lower BIC, but the quadratic model (M.3) showed the lower AICc. The LRT showed that M.2 was not statistically significantly different from M.3. However, M.2 was the most parsimonious and thus was chosen as best model. Also the effect size indices suggested a negligible preference for M.3 instead of M.2. Indeed, the ΔAICc suggested a very small difference M.2 and M.3 (1.66), the *W*_h_ of M.3 suggested that this model had 54% probability of being the best approximate model (*W*_h_ of M.2 was 32%), and the Eh suggested that M.3 had a weight of evidence almost two times (1.7) greater than M.2 of being the best approximate model (Table 3).

### Aggressive Behavior

Preliminary analyses (M.0) revealed that the variance related to the random intercept of the participants was equal to 14.70.

The null model with covariates (M.1) revealed a non-statistically significant effect of the interaction between sex and EXP-CVD19 (*b* = 1.333, *SE* = 2.518, *t* = 0.529, *p* = 0.598) or their main effects (sex: *b* = 0.250, *SE* = 1.376, *t* = 0.182, *p* = 0.856; EXP-CVD19: *b* = −1.507, *SE* = 1.802, *t* = −0.836, *p* = 0.405).

The linear model with covariates (M.2) revealed a non-statistically significant effect of the interaction between time and EXP-CVD19 (*b* = 0.670, *SE* = 0.788, *t* = 0.850, *p* = 0.396) or the two simple main effects (sex: *b* = 2.533, *SE* = 2.141, *t* = 1.183, *p* = 0.238; EXP-CVD19: *b* = −2.498, *SE* = 2.338, *t* = −1.069, *p* = 0.286). Moreover, the model revealed a non-statistically significant interaction effect between time and sex (*b* = −0.754, *SE* = 0.721, *t* = −1.045, *p* = 0.297) but only a statistically significant principal effect of time (*b* = 2.365, *SE* = 0.562, *t* = 4.221, *p* < 0.001).

The quadratic model with linear interaction (M.3) showed a non-statistically significant effect of the interaction between time and EXP-CVD19 (*b* = 0.670, *SE* = 0.785, *t* = 0.853, *p* = 0.394) or the two simple main effects (sex: *b* = 2.533, *SE* = 2.135, *t* = 1.186, *p* = 0.236; EXP-CVD19: *b* = −2.499, *SE* = 2.332, *t* = −1.071, *p* = 0.285). Moreover, the model revealed a non-statistically significant linear interaction effect between time and sex (*b* = −0.754, *SE* = 0.719, *t* = −1.049, *p* = 0.295) and the principal effect of time as a quadratic term (*b* = −0.580, *SE* = 0.402, *t* = −1.444, *p* = 0.150). Conversely, the model showed a statistically significant principal effect of time as a linear term (*b* = 5.265, *SE* = 2.085, *t* = 2.525, *p* = 0.012).

Finally, the quadratic model with all covariates interactions (M.4) showed a non-statistically significant effect of the interaction between time and EXP-CVD19, neither linear (*b* = 3.262, *SE* = 4.453, *t* = 0.733, *p* = 0.464) or quadratic (*b* = −0.519, *SE* = 0.877, *t* = −0.592, *p* = 0.555). In addition, M.4 showed also a non-statistically significant effect of the interaction between time and sex, neither linear (*b* = −2.320, *SE* = 4.077, *t* = −0.569, *p* = 0.570) nor quadratic (*b* = 0.313, *SE* = 0.830, *t* = 0.390, *p* = 0.670). Furthermore, also the simple main effects of sex (*b* = 4.099, *SE* = 4.546, *t* = 0.902, *p* = 0.368), EXP-CVD19 (*b* = −5.091, *SE* = 4.964, *t* = −1.026, *p* = 0.306), and the time both linear (*b* = 5.281, *SE* = 3.175, *t* = 1.663, *p* = 0.097) and quadratic (*b* = −0.583, *SE* = 0.625, *t* = −0.933, *p* = 0.352) revealed a non-statistically significant effect.

The comparison of the different multilevel growth curve models suggested that the linear model with covariates (M.2) showed the lower BIC and the lower AICc. The LRT showed that M.2 was statistically significantly different from M.1 (intercept model with covariates). However, although M.2 was not statistically significantly different from M.3, it was the most parsimonious—and thus, it was chosen as the best model. However, the effect size indices suggested a negligible preference for M.2. Indeed, the ΔAICc suggested a very small difference M.2 and M.3 (0.02), the *W*_h_ of M.2 suggested that this model had 47% probability of being the best approximate model (*W*_h_ of M.3 was 46%), and the *E*_h_ suggested that M.2 had a weight of evidence of 1.01 greater than M.3 of being the best approximate model (Table 3).

### Rule-Breaking Behavior

Preliminary analyses (M.0) revealed that the variance related to the random intercept of the participants was equal to 5.53.

The null model with covariates (M.1) revealed a non-statistically significant effect of the interaction between sex and EXP-CVD19 (*b* = −1.419, *SE* = 1.540, *t* = −0.922, *p* = 0.359) or the main effect of EXP-CVD19 (*b* = 0.246, *SE* = 1.102, *t* = 0.223, *p* = 0.824). Instead, the model shows a significant effect of sex (*b* = 3.235, *SE* = 0.842, *t* = 3.843, *p* < 0.001).

The linear model with covariates (M.2) revealed a non-statistically significant effect of the interaction between time and EXP-CVD19 (*b* = 0.263, *SE* = 0.548, *t* = 0.480, *p* = 0.632) or the two simple main effects (sex: *b* = 2.045, *SE* = 1.441, *t* = 1.419, *p* = 0.157; EXP-CVD19: *b* = −1.139, *SE* = 1.573, *t* = −0.724, *p* = 0.469). Moreover, the model revealed a non-statistically significant interaction effect between time and sex (*b* = 0.306, *SE* = 0.502, *t* = 0.610, *p* = 0.542) but a statistically significant principal effect of time (*b* = 1.223, *SE* = 0.391, *t* = 3.129, *p* = 0.002).

The quadratic model with linear interaction (M.3) showed a non-statistically significant effect of the interaction between time and EXP-CVD19 (*b* = 0.263, *SE* = 0.548, *t* = 0.480, *p* = 0.631) or the two simple main effects (sex: *b* = 2.045, *SE* = 1.440, *t* = 1.420, *p* = 0.156; EXP-CVD19: *b* = −1.139, *SE* = 1.573, *t* = −0.724, *p* = 0.469). Moreover, the model revealed a non-statistically significant linear interaction effect between time and sex (*b* = 0.306, *SE* = 0.502, *t* = 0.611, *p* = 0.542) as well as the principal effect of time: both linear (*b* = 2.009, *SE* = 1.455, *t* = 1.381, *p* = 0.168) and quadratic (*b* = −0.157, *SE* = 0.280, *t* = −0.561, *p* = 0.575).

Finally, the quadratic model with all covariates interactions (M.4) showed a non-statistically significant effect of the interaction between time and EXP-CVD19, neither linear (*b* = 0.408, *SE* = 3.110, *t* = 0.131, *p* = 0.896) nor quadratic (*b* = −0.029, *SE* = 10.612, *t* = −0.047, *p* = 0.962). In addition, M.4 showed also a non-statistically significant effect of the interaction between time and sex, neither linear (*b* = 1.757, *SE* = 2.847, *t* = 0.617, *p* = 0.538) nor quadratic (*b* = −0.290, *SE* = 0.560, *t* = −0.518, *p* = 0.605). Furthermore, also the simple main effects of sex (*b* = 0.594, *SE* = 3.151, *t* = 0.189, *p* = 0.851), EXP-CVD19 (*b* = −1.284, *SE* = 3.441, *t* = −0.373, *p* = 0.709), and time (linear term: *b* = 1.233, *SE* = 2.218, *t* = 0.556, *p* = 0.579; quadratic term: *b* = −0.001, *SE* = 0.436, *t* = 0.005, *p* = 0.996) revealed a non-statistically significant effect.

The comparison of the different multilevel growth curve models suggested that the linear model with covariates (M.2) showed the lower BIC and the lower AICc. The LRT showed that M.2 was statistically significantly different from M.1 (intercept model with covariates). However, despite that M.2 was not statistically significantly different from M.3, it was the most parsimonious—and thus, it was chosen as the best model. However, the effect size indices suggested a small preference for M.2. Indeed, the ΔAICc suggested a small difference M.2 and M.3 (1.78), the *W*_h_ of M.2 indicates that this model had 68% probability of being the best approximate model, and the *E*_h_ recommend that M.2 had a weight of evidence more than two times (2.24) greater than M.3 of being the best approximate model (Table 3).

### Intrusive Behavior

Preliminary analyses (M.0) revealed that the variance related to the random intercept of the participants was equal to 2.81.

The null model with covariates (M.1) revealed a non-statistically significant effect of the interaction between sex and EXP-CVD19 (*b* = 0.801, *SE* = 1.339, *t* = 0.598, *p* = 0.551) or their main effects (sex: *b* = 1.059, *SE* = 0.732, *t* = 1.446, *p* = 0.151; EXP-CVD19: *b* = −0.393, *SE* = 0.958, *t* = −0.410, *p* = 0.683).

The linear model with covariates (M.2) revealed a non-statistically significant effect of the interaction between time and EXP-CVD19 (*b* = 0.165, *SE* = 0.509, *t* = 0.324, *p* = 0.746) or the two simple main effects (sex: *b* = 1.282, *SE* = 1.317, *t* = 0.973, *p* = 0.331; EXP-CVD19: *b* = −0.395, *SE* = 1.439, *t* = −0.275, *p* = 0.784). Moreover, the model revealed a non-statistically significant interaction effect between time and sex (*b* = 0.006, *SE* = 0.466, *t* = 0.014, *p* = 0.989) as well as the principal effect of time (*b* = −0.685, *SE* = 0.363, *t* = −1.88, *p* = 0.060).

The quadratic model with linear interaction (M.3) showed a non-statistically significant effect of the interaction between time and EXP-CVD19 (*b* = 0.165, *SE* = 0.509, *t* = 0.324, *p* = 0.746) or the two simple main effects (sex: *b* = 1.281, *SE* = 1.317, *t* = 0.973, *p* = 0.331; EXP-CVD19: *b* = −0.395, *SE* = 1.438, *t* = −0.275, *p* = 0.784). Moreover, the model revealed a non-statistically significant linear interaction effect between time and sex (*b* = 0.006, *SE* = 0.466, *t* = 0.014, *p* = 0.989) as well as the principal effect of time: both linear (*b* = −0.557, *SE* = 1.352, *t* = −0.412, *p* = 0.681) and quadratic (*b* = −0.025, *SE* = 0.260, *t* = −0.099, *p* = 0.921).

Finally, the quadratic model with all covariates interactions (M.4) showed a non-statistically significant effect of the interaction between time and EXP-CVD19, neither linear (*b* = 1.431, *SE* = 2.889, *t* = 0.495, *p* = 0.621) nor quadratic (*b* = −0.253, *SE* = 0.568, *t* = −0.445, *p* = 0.656). In addition, M.4 showed also a non-statistically significant effect of the interaction between time and sex, neither linear (*b* = 1.481, *SE* = 2.645, *t* = 0.560, *p* = 0.576) nor quadratic (*b* = −0.295, *SE* = 0.521, *t* = −0.566, *p* = 0.572). Furthermore, also the simple main effects of sex (*b* = −0.192, *SE* = 2.918, *t* = −0.066, *p* = 0.947), EXP-CVD19 (*b* = −1.662, *SE* = 3.187, *t* = −0.521, *p* = 0.602), and time (linear term: *b* = −1.680, *SE* = 2.060, *t* = −0.815, *p* = 0.415; quadratic term: *b* = 0.199, *SE* = 0.406, *t* = 0.490, *p* = 0.624) revealed a non-statistically significant effect.

The comparison of the different multilevel growth curve models provided unclear results. Indeed, the intercept-only model (without covariates—M.0) showed the lower BIC, but the linear model (M.2) showed the lower AICc. The LRT showed that M.0 was not statistically significantly different from M.1 (intercept model with covariates) but M.2 was statistically significantly different from M.1. In addition, the effect size indices suggested a negligible preference for M.2. Indeed, the ΔAICc suggested a very small difference M.2 and M.0 (1.44), the *W*_h_ of M.2 suggested that this model had 47% probability of being the best approximate model (*W*_h_ of M.0 was 23%), and the *E*_h_ suggested that M.2 had a weight of evidence two times (2.05) greater than M.0 of being the best approximate model (Table 3).

### Internalizing Broadband Scale

Preliminary analyses (M.0) revealed that the variance related to the random intercept of the participants was equal to 16.37.

The null model with covariates (M.1) revealed a non-statistically significant effect of the interaction between sex and EXP-CVD19 (*b* = 0.514, *SE* = 3.224, *t* = −0.159, *p* = 0.874) or their main effects (sex: *b* = −0.059, *SE* = 1.762, *t* = −0.033, *p* = 0.973; EXP-CVD19: *b* = −0.690, *SE* = 0.307, *t* = −0.299, *p* = 0.766).

The linear model with covariates (M.2) revealed a statistically significant effect of the interaction between time and EXP-CVD19 (*b* = 2.540, *SE* = 1.062, *t* = 2.392, *p* = 0.017) and the EXP-CVD19 simple main effect (*b* = −7.304, *SE* = 3.106, *t* = −2.351, *p* = 0.019). Moreover, the model revealed a non-statistically significant interaction effect between time and sex (*b* = −1.269, *SE* = 0.972, *t* = −1.305, *p* = 0.193) and the sex simple main effect (*b* = 2.961, *SE* = 2.844, *t* = 1.041, *p* = 0.298). Only the main effect of time (*b* = 4.061, *SE* = 0.757, *t* = 5.636, *p* < 0.001) became statistically significant.

The quadratic model with linear interaction (M.3) showed a statistically significant effect of the interaction between time and EXP-CVD19 (*b* = 2.540, *SE* = 1.051, *t* = 2.417, *p* = 0.016) and the simple main effects of EXP-CVD19 (*b* = −7.304, *SE* = 3.083, *t* = −2.369, *p* = 0.018). However, the model revealed a non-statistically significant linear interaction effect between time and sex (*b* = −1.269, *SE* = 0.962, *t* = −1.319, *p* = 0.188) and the sex simple main effect (*b* = 2.961, *SE* = 2.822, *t* = 3.827, *p* < 0.001). The effects of time both linear (*b* = 10.685, *SE* = 2.792, *t* = 3.827, *p* < 0.001) and quadratic (*b* = −1.325, *SE* = 0.538, *t* = −2.463, *p* = 0.014) became statistically significant.

Finally, the quadratic model with all covariates interactions (M.4) showed a non-statistically significant effect of the interaction between time and EXP-CVD19, neither linear (*b* = 9.509, *SE* = 5.950, *t* = 1.598, *p* = 0.111) nor quadratic (*b* = −1.394, *SE* = 1.171, *t* = −1.190, *p* = 0.325). In addition, M.4 showed also a non-statistically significant effect of the interaction between time and sex, neither linear (*b* = −4.910, *SE* = 5.448, *t* = −0.901, *p* = 0.368) nor quadratic (*b* = 0.728, *SE* = 1.073, *t* = 0.679, *p* = 0.498). Furthermore, also the simple main effects of sex (*b* = −6.601, *SE* = 6.058, *t* = 1.090, *p* = 0.277) and time (quadratic term: *b* = −1.275, *SE* = 0.835, *t* = −1.527, *p* = 0.128) revealed a non-statistically significant effect. The main effects of EXP-CVD19 (*b* = −14273, *SE* = 6.616, *t* = −2.157, *p* = 0.031) and the time as a linear term (*b* = 10.439, *SE* = 4.243, *t* = 2.460, *p* = 0.014) became statistically significant.

The comparison of the different multilevel growth curve models suggested that the quadratic model with linear covariates interaction (M.3) showed the lower BIC and the lower AICc. The LRT showed that M.3 was statistically significantly different from M.2 (linear model with covariates). However, the LRT suggested that M.3 was not statistically significantly different from M.4, but it was more parsimonious—and thus, M.3 was chosen as the best model. However, the effect size indices suggested a small preference for M.3. Indeed, the ΔAICc suggested a small difference M.3 and M.4 (2.38), the *W*_h_ of M.3 suggested that this model had 69% probability of being the best approximate model, and the *E*_h_ suggested that M.3 had a weight of evidence more than three times (3.29) greater than M.4 of being the best approximate model (Table 3).

### Externalizing Broadband Scale

Preliminary analyses (M.0) revealed that the variance related to the random intercept of the participants was equal to 5.82.

The null model with covariates (M.1) revealed a non-statistically significant effect of the interaction between sex and EXP-CVD19 (*b* = 0.013, *SE* = 2.269, *t* = 0.006, *p* = 0.996) or their main effects (sex: *b* = 2.264, *SE* = 1.240, *t* = 1.826, *p* = 0.071; EXP-CVD19: *b* = −0.315, *SE* = 1.624, *t* = −0.194, *p* = 0.847).

The linear model with covariates (M.2) revealed a non-statistically significant effect of the interaction between time and EXP-CVD19 (*b* = 1.131, *SE* = 0.867, *t* = 1.303, *p* = 0.193) or the two simple main effects (sex: *b* = 3.319, *SE* = 2.241, *t* = 1.481, *p* = 0.139; EXP-CVD19: *b* = −3.135, *SE* = 2.448, *t* = −1.281, *p* = 0.201). Moreover, the model revealed a non-statistically significant interaction effect between time and sex (*b* = −0.420, *SE* = 0.794, *t* = −0.529, *p* = 0.597). Only the main effect of time (*b* = 2.266, *SE* = 0.619, *t* = 3.662, *p* < 0.001) became statistically significant.

The quadratic model with linear interaction (M.3) showed a non-statistically significant effect of the interaction between time and EXP-CVD19 (*b* = 1.130, *SE* = 0.864, *t* = 1.308, *p* = 0.192) or the two simple main effects (sex: *b* = 3.319, *SE* = 2.235, *t* = 1.485, *p* = 0.138; EXP-CVD19: *b* = −3.135, *SE* = 2.441, *t* = −1.284, *p* = 0.199). Moreover, the model revealed a non-statistically significant linear interaction effect between time and sex (*b* = −0.420, *SE* = 0.792, *t* = −0.531, *p* = 0.596) as well as the principal effect of time as a quadratic term (*b* = −0.644, *SE* = 0.442, *t* = −1.456, *p* = 0.146). Only the main effect of time as a linear term (*b* = −5.487, *SE* = 2.296, *t* = 2.389, *p* = 0.017) became statistically significant.

Finally, the quadratic model with all covariates interactions (M.4) showed a non-statistically significant effect of the interaction between time and EXP-CVD19, neither linear (*b* = 4.135, *SE* = 4.905, *t* = 0.943, *p* = 0.400) nor quadratic (*b* = −0.601, *SE* = 0.966, *t* = −0.622, *p* = 0.534). In addition, M.4 showed also a non-statistically significant effect of the interaction between time and sex, neither linear (*b* = 0.965, *SE* = 4.492, *t* = 0.215, *p* = 0.830) nor quadratic (*b* = −0.277, *SE* = 0.884, *t* = −0.313, *p* = 0.754). Furthermore, also the simple main effects of sex (*b* = 1.934, *SE* = 4.953, *t* = 0.390, *p* = 0.696), EXP-CVD19 (*b* = −6.140, *SE* = 5.410, *t* = −1.135, *p* = 0.258), and time (linear term: *b* = 3.889, *SE* = 3.498, *t* = 1.112, *p* = 0.267, and quadratic term: *b* = −0.325, *SE* = 0.689, *t* = −0.472, *p* = 0.638) revealed a non-statistically significant effect.

The comparison of the different multilevel growth curve models provided unclear results. Indeed, the linear model (M.2) showed the lower BIC, but the quadratic model (M.3) showed the lower AICc. The LRT showed that M.3 was not statistically significantly different from M.2. However, despite that M.2 was not statistically significantly different from M.3, it was the most parsimonious—and thus, it was chosen as the best model. However, the effect size indices suggested a negligible preference for M.3. Indeed, the ΔAICc suggested a very small difference M.3 and M.2 (0.02), the *W*_h_ of M.3 suggested that this model had 47% probability of being the best approximate model (*W*_h_ of M.2 was 46%), and the *E*_h_ suggested that M.3 had a weight of evidence of 1.01 greater than M.2 of being the best approximate model (Table 3).

### Personal Strengths

Preliminary analyses (M.0) revealed that the variance related to the random intercept of the participants was equal to 1.18.

The null model with covariates (M.1) revealed a non-statistically significant effect of the interaction between sex and EXP-CVD19 (*b* = −0.248, *SE* = 0.792, *t* = −0.313, *p* = 0.755) or their main effects (sex: *b* = −0.154, *SE* = 0.433, *t* = −0.357, *p* = 0.722; EXP-CVD19: *b* = 0.499, *SE* = 0.567, *t* = 0.880, *p* = 0.381).

The linear model with covariates (M.2) revealed a non-statistically significant effect of the interaction between time and EXP-CVD19 (*b* = 0.066, *SE* = 0.274, *t* = 0.242, *p* = 0.809) or the two simple main effects (sex: *b* = 0.228, *SE* = 0.724, *t* = 0.315, *p* = 0.752; EXP-CVD19: *b* = 0.206, *SE* = 0.791, *t* = 0.261, *p* = 0.794). Moreover, the model revealed a non-statistically significant interaction effect between time and sex (*b* = −0.182, *SE* = 0.250, *t* = −0.729, *p* = 0.466). Only the main effect of time (*b* = −0.513, *SE* = 0.195, *t* = −2.629, *p* = 0.009) became statistically significant.

The quadratic model with linear interaction (M.3) showed a non-statistically significant effect of the interaction between time and EXP-CVD19 (*b* = 0.066, *SE* = 0.273, *t* = 0.242, *p* = 0.809) or the two simple main effects (sex: *b* = 0.228, *SE* = 0.723, *t* = 0.316, *p* = 0.752; EXP-CVD19: *b* = 0.206, *SE* = 0.789, *t* = −0.261, *p* = 0.794). Moreover, the model revealed a non-statistically significant linear interaction effect between time and sex (*b* = −0.182, *SE* = 0.250, *t* = −0.730, *p* = 0.466) as well as the principal effect of time: both linear (*b* = −1.157, *SE* = 0.726, *t* = −1.594, *p* = 0.112) and quadratic (*b* = 0.128, *SE* = 0.139, *t* = 0.921, *p* = 0.358).

Finally, the quadratic model with all covariates interactions (M.4) showed a non-statistically significant effect of the interaction between time and EXP-CVD19, neither linear (*b* = 0.820, *SE* = 1.549, *t* = 0.529, *p* = 0.597) nor quadratic (*b* = −0.150, *SE* = 0.305, *t* = −0.494, *p* = 0.621). In addition, M.4 showed also a non-statistically significant effect of the interaction between time and sex, neither linear (*b* = −1.314, *SE* = 1.418, *t* = −0.926, *p* = 0.355) nor quadratic (*b* = 0.226, *SE* = 0.279, *t* = 0.810, *p* = 0.418). Moreover, also the simple main effects of sex (*b* = 1.360, *SE* = 1.572, *t* = 0.865, *p* = 0.388), EXP-CVD19 (*b* = −0.547, *SE* = 1.717, *t* = −0.319, *p* = 0.750), and time (linear term: *b* = −0.811, *SE* = 1.104, *t* = −0.734, *p* = 0.463; quadratic term: *b* = 0.059, *SE* = 0.217, *t* = 0.274, *p* = 0.784) revealed a non-statistically significant effect.

The comparison of the different multilevel growth curve models suggested that the linear model with covariates (M.2) showed the lower BIC and the lower AICc. The LRT showed that M.2 was statistically significantly different from M.1 (null model with covariates). However, the LRT suggested M.2 was not statistically significantly different from M.3, but it was more parsimonious—and thus, M.3 was chosen as the best model. However, the effect size indices suggested a small preference for M.2. Indeed, the ΔAICc suggests a small difference M.2 and M.3 (1.25), the *W*_h_ of M.2 indicates that this model had 61% probability of being the best approximate model (*W*_h_ of M.3 was 33%), and the *E*_h_ suggested that M.2 had a weight of evidence almost two times (1.87) greater than M.3 of being the best approximate model (Table 3).

## Discussion

As stated above, in addition to being a public physical health emergency, the COVID-19 pandemic also implies a global mental health emergency that may have a potential traumatic nature and provoke complex emotional responses that could negatively affect individual and collective mental health (Jakovljevi et al., 2020; Masiero et al., 2020). Therefore, this global pandemic constantly requires researchers and professionals to monitor and assess the current mental health situation, in order to plan and develop efficiency-driven strategies aimed to reduce its negative psychological impacts.

This study assessed and monitored Italian young adults’ mental health status during the firsts 4 weeks of lockdown imposed by the government during the COVID-19 outbreak, from March 16 to April 16. To the authors’ knowledge, this is the first study specifically focused on young adults’ mental health status during COVID-19 quarantine, both in Italy and worldwide. A longitudinal panel design was carried out in order to assess *Internalizing* and *Externalizing problems* on 97 Italian young adults living in the Campania region, Southern Italy. A GCA (Jackson et al., 2018) was performed to monitor the changes during the first 4 weeks of quarantine.

First of all, in line with the global trend reported by previous studies carried out on the general population (Cao et al., 2020; Huang and Zhao, 2020; Qiu et al., 2020; Rossi R. et al., 2020), this study confirmed the negative behavioral and emotional responses provoked by COVID-19 quarantine and also highlighted the high vulnerability of young adults in developing psychological distress.

Comparing the *Internalizing* and *Externalizing domains*, the results showed an analogous increase for both areas from T1 to T4, even though higher rates of internalizing manifestations were registered. Specifically, the growth curve modeling highlighted that, within the Internalizing problems area, the levels of *Anxiety/Depression*, *Withdrawal*, and *Somatic Complaints* overall increased from T1 to T4, showing an increase while the lockdown measures were in place. In this context, in line with results obtained on medical health workers (Zhang et al., 2020), having experienced a closeness with a COVID-19-infected relative or friend resulted in an increase of somatic complaints. Similarly, within the Externalizing problems area, the levels of *Aggressive Behavior* and *Rule Breaking Behavior* increased from T1 to T4. Among the *Internalizing domains*, youth reported clinical-level symptoms of anxiety and depression. According to the recent review on the psychological impact of quarantine (Rajkumar, 2020), anxiety as well as depressive symptomatology was the most common. Furthermore, the results showed that *Withdrawal* level was above the normal threshold. This finding could be related to the specific situation of quarantine and the impossibility to engage in social behaviors due to the lockdown. Indeed, the physical distance can intensify feelings of loneliness that in turn trigger intense anxiety (Boffo et al., 2012; Banerjee and Rai, 2020; Rossi A. et al., 2020).

If, broadly, the results obtained confirmed the general detrimental effects of social isolation due to epidemics on young adults’ mental health (Hawryluck et al., 2004; Tucci et al., 2017; Qiu et al., 2020; Wang et al., 2020), some brief reflections need to be outlined about the specificities of young adults’ condition. Indeed, young adults live a specific transition period in which their identity development process is based and founded on continuous affective investments on social and extra-familiar relationships (Sica et al., 2018). In this context, the lockdown measures may be interpreted as a forced regression that triggers negative mental health outcomes even more. Within the range from T1 to T4, higher levels of *Internalizing* and *Externalizing* problems were registered at T3, whereas a sort of stabilization from T3 to T4 emerged. The peak reported at T3 probably indicated a sort of gradual cognitive and emotional recognition experienced from young adults about the seriousness of the pandemic, which increased feelings of anxiety, depression and worry, and irritability and anger. Regarding the stabilization of both internalizing and externalizing problems between T3 and T4, these findings might need to be interpreted in relation to the specific historical context of the COVID-19 pandemic in Italy. Specifically, T4 corresponded to the week from April 16 to 12 in which a double attitude was observed in Italy. On the one hand, despite the lockdown, the Italian “Civil Protection” continued to alert the general population about the very high levels of contagions; on the other hand, in that period, Italians also started to receive the first information about the so-called “Phase 2,” which followed the forced lockdown. It might be hypothesized that the high levels of viral load continued to worry participants, even though the closeness to Phase 2 assumed a sort of protective function regarding an eventual mental health worsening.

In correspondence to the increase of mental health distress, the results also showed a gradual decrease of participants’ perception of their personal strengths, suggesting the need for researchers to strengthen individual’s psychological resources in order to mediate the individual reaction to the COVID-19 pandemic (Di Giuseppe et al., 2020).

In conclusion, regarding gender differences, a significant increase of the levels of *Anxiety/Depression* from T1 to T2 and, to a lesser extent, from T2 to T3 in males than the females emerged. These findings were in line with previous studies that pointed out higher symptoms of anxiety and depression in condition of social isolations in boys than girls (Troop-Gordon and Ladd, 2005; Derdikman-Eiron et al., 2011). The results reported no other statistically significant differences between sex. These findings seemed to be in opposition with the recent studies that have investigated the impact of COVID-19 on mental health and highlighted a higher vulnerability for women to develop negative mental health outcomes, as compared with men (Qiu et al., 2020; Rossi R. et al., 2020). In the context of gender studies, a wide range of recent literature tended to connect these results to the reinforced gender inequalities promoted by the lockdown measures. According to these studies (Adams-Prassl et al., 2020; Béland et al., 2020; Etheridge and Spantig, 2020), in fact, during the lockdown measures, the increase of unemployment rates as well as the commitment into the domestic work and in the management of children has represented a high risk factor for women, compared with men. Within the same interpretation field, the lack of significant gender differences as emerged by the results might be correlated to the same nature of the sample, which mostly involved university students who probably were involved in the same challenges and tasks and did not experienced greater or smaller efforts connected to specific gender roles, such as to outline differences.

The present study is not free from limitations. First of all, the number of participants should be increased in future studies, and the results need to be replicated in other geographical areas to determine their generalizability. Furthermore, the sample was only composed of university students who came from the Campania region in Southern Italy where the COVID-19 outbreak has been taken more under control. To assess the mental health of young people during the quarantine, only a self-report measure was used. Consequently, the data may be influenced by a reporting bias (e.g., social desirability). Moreover, despite the longitudinal panel, the study is an observational study. In this sense, experimental manipulations and a control group are lacking. Future researches need to extend the young adults’ mental health assessment to other Italian regions, taking into consideration that in the South of Italy, where the study was carried out, the COVID-19 outbreak has been taken moderately and was under control, compared with the North. Higher levels of distress might be hypothesized in places where very high numbers of losses and deaths have been registered. Moreover, the present study investigated the internalizing and externalizing problems as individual responses to COVID-19 pandemic; further investigations to measure the traumatic symptomatology and the characteristics of post-traumatic effects caused by such stressful events are needed (Troisi, 2018; Margherita and Tessitore, 2019). Follow-up investigations are also needed. Considering the high levels of *Withdrawal* that emerged from the results, future investigations should explore the function and the role played by virtual environments and e-communities during pandemic in-depth, taking into account the roles played by the online environments and by the use of social media in terms of both risks and protective functions (Faccio et al., 2019; Gargiulo and Margherita, 2019; Margherita and Gargiulo, 2018; Procentese et al., 2019; Boursier et al., 2020). In this sense, future investigations might be also directed to investigate the changes in the dynamics of social and love relationships (Mannarini et al., 2013, 2017a; Balottin et al., 2017; Margherita et al., 2018) as well as the role of social support (Ratti et al., 2017) post-lockdown and post-pandemic. In conclusion, recognizing the fundamental value of qualitative investigations to shed light on the inner aspects and subjective meanings of personal experiences is also vital (Margherita et al., 2017; Tessitore and Margherita, 2019; Tessitore et al., 2019; Felaco and Parola, 2020; Parola, 2020; Parola and Felaco, 2020; Tessitore and Margherita, 2020). These are much needed actions in order to develop an in-depth understanding of the emotional and affective dimensions connected to the experience of the COVID-19 pandemic, as well as possible risk and protective factors for mental health.

In conclusion, the present study could contribute to the ongoing debate concerning the psychological impact of the COVID-19 emergency, helping to develop efficient and person-centered intervention projects able to take care of young adults’ mental health in the medium and long terms, understanding their specific needs and susceptibilities (Benedetto et al., 2018; Parola and Donsì, 2018, Parola and Donsì, 2019; Fusco et al., 2019). This is even more urgent considering that despite the distressing and prolonged situation, a significant number of people avoid seeking psychological help (Rossi and Mannarini, 2019). On the one hand, some of these people may be reluctant to seek professional help due to the associated stigma (Mannarini et al., 2017b, 2018, 2020; Faccio et al., 2019; Mannarini and Rossi, 2019). On the other hand, some individuals may deny the problem, leading them to think that it will probably resolve itself naturally (Sareen et al., 2007; Rossi Ferrario et al., 2019; Rossi Ferrario and Panzeri, 2020), thus choosing to manage the psychological issue on their own (Wilson and Deane, 2012).

## Data Availability Statement

The datasets generated in this article are not readily available because to ensure the privacy of the participants. Requests to access the datasets should be directed to AP, anna.parola@un ina.it.

## Ethics Statement

The studies involving human participants were reviewed and approved by the Ethical Committee of Psychological Research of University of Naples Federico II and was carried out in accordance with the American Psychological Association rules. The patients/participants provided their written informed consent to participate in this study.

## Author Contributions

AP developed the theoretical framework of the present study, designed the study, and developed the methodological approach. AR performed all the analyses and designed tables and figures. FT and GT led the literature search and interpretation of data. SM critically revised the manuscript. All authors read and approved the final version of the work.

## Conflict of Interest

The authors declare that the research was conducted in the absence of any commercial or financial relationships that could be construed as a potential conflict of interest.
